# Human Cutaneous Anthrax, Georgia 2010–2012

**DOI:** 10.3201/eid2002.130522

**Published:** 2014-02

**Authors:** Ian Kracalik, Lile Malania, Nikoloz Tsertsvadze, Julietta Manvelyan, Lela Bakanidze, Paata Imnadze, Shota Tsanava, Jason K. Blackburn

**Affiliations:** University of Florida, Gainesville, Florida, USA (I. Kracalik, J.K. Blackburn);; National Center for Disease Control and Public Health of Georgia, Tbilisi, Georgia (L. Malania, N. Tsertsvadze, J. Manvelyan, P. Imnadze, S. Tsanava);; Agrarian University of Georgia, Tbilisi (L. Bakanidze)

**Keywords:** anthrax, cutaneous anthrax, zoonoses, Georgia, bacteria, Bacillus anthracis, livestock

## Abstract

We assessed the occurrence of human cutaneous anthrax in Georgia during 2010–-2012 by examining demographic and spatial characteristics of reported cases. Reporting increased substantially, as did clustering of cases near urban centers. Control efforts, including education about anthrax and livestock vaccination, can be directed at areas of high risk.

Anthrax, a zoonosis caused by the bacterium *Bacillus anthracis*, is associated with rural areas or agricultural production (*1*–*3*). Human infections often result directly from contact with infected livestock or contaminated animal materials (*2*). Although several countries have implemented successful control strategies, anthrax remains a reemerging threat to public health in areas with weak health systems. After the Soviet Union collapsed, the country of Georgia underwent funding cuts to public health and animal health infrastructure, limiting disease management (*2*). Human anthrax cases more than tripled in Georgia after Soviet governance discontinued; 118 cases were reported during 1991–1996, compared with 36 during 1985–1990 (*4*,*5*). Recent reports indicating a worsening anthrax situation in Georgia have raised concern about spread of anthrax to new areas (*5*,*6*). To assess this situation, we analyzed demographic risk factors and characterized spatial patterns of human cutaneous anthrax (HCA) in Georgia.

## The Study

Anthrax is nationally reportable in Georgia. We analyzed passive surveillance data on HCA cases reported to the National Center for Disease Control and Public Health (Tbilisi, Georgia) during 2010–2012. Case investigation forms included patients’ community of residence, age, and sex. Self-reported source of infection was ascertained from categorical responses that included butchering/slaughtering cattle, handling/preparing meat, performing field work/harvesting crops, processing hair/wool, receiving an insect bite, unknown, or other. We also obtained information about regional-level anthrax cases in livestock (www.oie.int/wahis_2/public/wahid.php/diseaseinformation/statusdetail). HCA cases and incidence per million persons were plotted over time with livestock cases. To estimate changes in HCA risk over time, we compared data for the study period with data for 2007–2009 by using a cumulative incidence ratio (total cases/median year population). We calculated incidence risk ratios for demographic characteristics by using a negative binomial regression (Technical Appendix).

Data were anonymized and aggregated to each case-patient’s community of residence and mapped in a geographic information system. We extrapolated current population from the 2002 census using the United Nations medium variant population growth projections (http://data.un.org/). Cumulative incidences per 10,000 population were calculated for each community (total cases/median year population). National and community population data were obtained from GeoStat (http://geostat.ge).

We adjusted for community population heterogeneity using Empirical Bayes smoothing estimates of the cumulative incidence (Technical Appendix). Kernel density estimation was used to map the cumulative incidence risk/km^2^ (Technical Appendix). To identify community-level HCA spatial clustering, we used the Poisson model in SaTScan (*7*) with a maximum cluster size of 25% of the population at risk. We compared the proportion of urban and rural communities inside and outside clusters using a χ^2^ analysis (PROC FREQ, SAS Institute, Cary, NC, USA).

**Figure 1:**
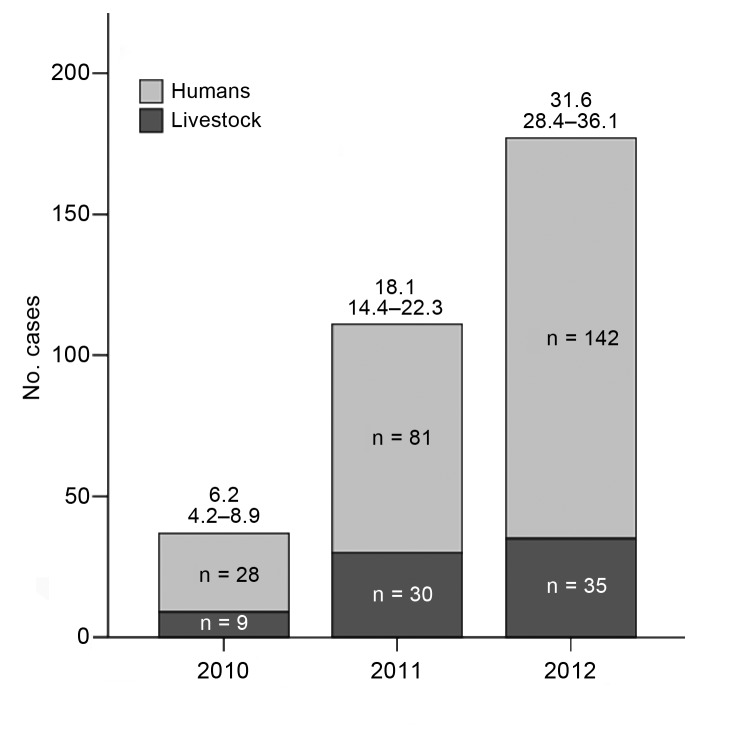
Total number of human cutaneous anthrax cases (light gray) and livestock cases (dark gray), Georgia, 2010–2012. Incidence rates (IRs) (95% CIs) of human cutaneous anthrax per million population are displayed above the bars.

During 2010–2012, a total of 251 HCA cases and 74 livestock cases (38 cattle, 1 pig, 4 sheep/goats, 1 horse) were reported (Figure 1). HCA cases peaked in 2012 (142 cases), corresponding to a national incidence of 31.7 per million population (95% CI 28.4–36.1). Compared with 2007–2009 (133 cases), the period studied had a higher risk for HCA (cumulative incidence ratio 1.89 [95% CI 1.64–2.33], p<0.01).

Cases occurred predominantly in males (209 [84%]) (Table 1). Butchering/slaughtering cattle as a source of infection was more common among male patients (143 [68%]); processing/handling meat was more common among female patients (28 [68%]). Case-patients’ median age was 43 years [range 5–79]. The adjusted incidence risk ratios from the negative binomial model showed a stronger association of risk in males (4.95 [95% CI 2.91–8.42]) than in females (Table 2). Persons 50–64 years of age were at greater risk than were persons in all other age groups. Risk did not differ between case-patients who processed/handled meat and those who slaughtered/butchered cattle.

**Table 1:** Characteristics of human cutaneous anthrax, Georgia, 2010–2012

Characteristic	Male case-patients			Female case-patients	
	No., n = 209	Population*		No., n = 42	Population*
Age, y					
5–19	14	522,736		2	506,785
20–34	57	458,998		8	480,276
35–49	62	443,820		12	502,732
50–64	61	292,713		15	361,370
65–79	15	196,171		5	286,839
Self-reported infection source					
Slaughtering cattle	143			3	
Processing meat	39			28	
Field work/sowing and harvesting crops	20			7	
Unknown	7			3	

*Population estimates were obtained from the Georgian State Statistical Office (GeoStat, http://geostat.ge) and are based on median year population totals for the study period.

**Table 2:** Results of the negative binomial regression model examining risk factors for human cutaneous anthrax Georgia

Patient characteristic	IRR*		95% CI†	p value
	Univariate	Adjusted		
Age, y				
5–19	0.12	0.11	0.05–0.26	<0.01
20–34	0.57	0.46	0.23–0.91	0.03
35–49	0.65	0.58	0.30–1.14	0.11
50–64	Referent	Referent		–
65–79	0.37	0.36	0.16–0.78	0.01
Sex				
F	Referent	Referent		–
M	5.75	4.95	2.91–8.42	<0.01
Self-reported infection source				
Slaughtering/butchering cattle	Referent	Referent		–
Processing/handling meat	0.45	0.75	0.40–1.39	0.36
Field work/sowing and harvesting crops	0.18	0.26	0.13–0.51	<0.01
Unknown	0.07	0.09	0.04–0.20	<0.01

*χ^2^ goodness-of-fit test indicated the model fit the data (df = 31, χ^2^ = 40.71, p = 0.11). IRR, incidence risk ratio. †Wald 95% CIs.

**Figure 2:**
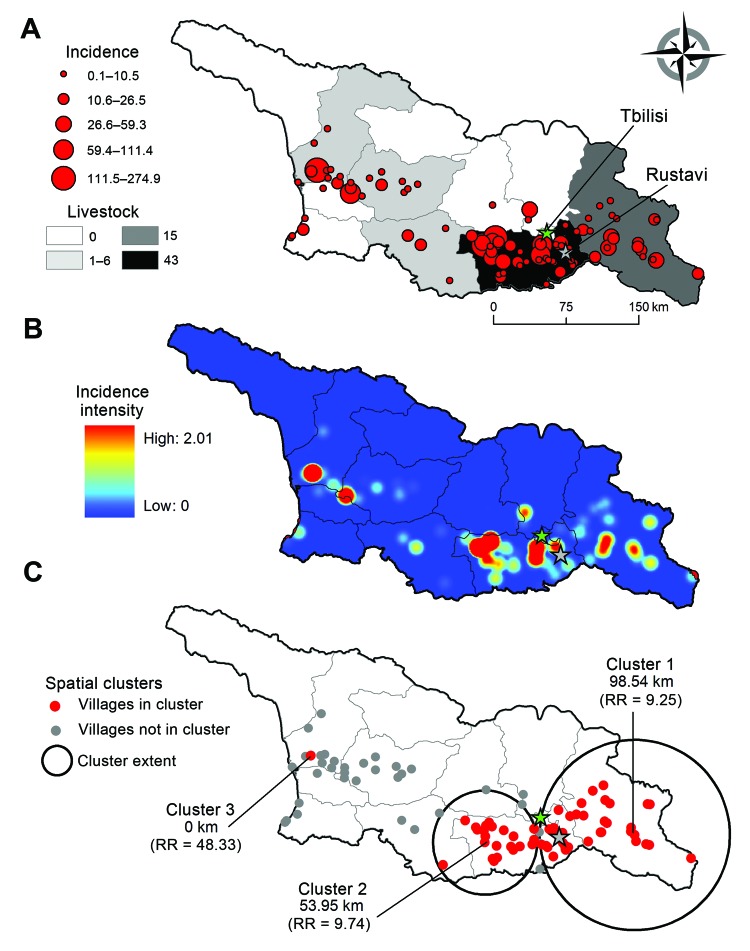
A) Empirical Bayes Smoothing cumulative incidence (per 10,000 population) of human cutaneous anthrax at the community level, Georgia, 2010–2012. Green star indicates the location of the capital, Tbilisi; gray star indicates the fourth most populous city, Rustavi. The total number of livestock cases during the study period is shown by region. B) Risk surface representing the estimated smoothed cumulative incidence per square kilometer. C) Spatial clustering of human cutaneous anthrax cases as determined by using SaTScan (*7*). RR, risk ratio.

Empirical Bayes smoothed incidence per 10,000 population ranged from 0.11 (95% CI 0.03–0.37) to 213.8 (95% CI 72.8–552.6) (Figure 2, panel A). The kernel density analysis identified a higher risk per squared kilometer in the southeast and west (Figure 2, panel B). We identified 3 spatial clusters of high risk; the clusters comprised 58 communities with 197 cases (Figure 2, panel C). Cumulative incidence inside the clusters was 12.8/10,000 population compared with 0.96/10,000 population outside the clusters. The proportion (χ^2^ = 0.65, p = 0.4) of urban and rural communities (22 and 36, respectively) within clusters did not differ from the proportion outside clusters (urban: 14; rural: 24). Clusters 1 and 2 in the southeast bordered the densely populated urban area of the capital (Tbilisi) and were associated with grazing lands, whereas cluster 3 bordered the urban area of Poti in the west and was associated with a higher percentage of croplands.

## Conclusions

Despite an apparent decline in HCA cases worldwide (*8*), incidence in Georgia has increased and is now comparable with that in Turkey, where the disease is hyperendemic (*1*,*9*,*10*). Our analysis identified clusters of communities that were of historical importance for HCA risk and new communities that represent areas of (re)emergence (*6*).

Our findings indicate that HCA cases were concentrated within specific areas associated with agriculture and in close proximity to urban centers (Figure 2), consistent with research linking urban livestock trading centers and markets to outbreaks and higher rates of HCA (*5*,*9*–*11*). Sick and dying animals are often slaughtered and brought to market quickly to mitigate economic losses, thereby exacerbating exposure risk while limiting livestock reporting (*10*,*11*). HCA is primarily associated with rural, agrarian areas (*2*,*3*,*12*), but we found no difference in the proportion of urban and rural communities in high-risk clusters. This association with urban zones suggests reliance on these areas for commerce, coupled with increased agricultural employment from 25% of the population in 1990 to ≈55% in 2010 (*5*,*13*). One hypothesis is that contaminated meat is brought into urban areas and sold at informal meat markets that because of fiscal constraints have little to no regulation.

Cases were predominantly linked to contact with infected livestock or meat, as observed elsewhere (*2*,*3*,*9*,*11*). Field work was a self-reported source of infection in 27 (10.7%) cases. This risk factor is not well documented and might reflect recall bias or unwillingness to admit to slaughtering infected animals. Our findings that males were at higher risk than females for HCA, as reported elsewhere (*2*,*14*) might reflect occupational exposures or gender roles, with males slaughtering/butchering livestock and females more involved in preparing meat (*14*). In contrast, no gender differences were reported in Turkey or Kazakhstan (*3*,*9*). Although age was not a risk factor in other regions (*2*,*3*), persons 50–64 years of age in this study were at higher risk for infection (Table 2), reflecting changing sociocultural practices related to an agrarian lifestyle.

Although the true cause of the increase in HCA cases is unknown, it is probably due to a combination of factors related to decreased public health funding and agricultural reform resulting in a shift from collectivization to private ownership (*1*,*5*,*13*). These factors, coupled with limited veterinary control and the cessation of compulsory livestock vaccination, have played plausible roles in the increased incidence of HCA. Livestock cases were out of sync temporally and spatially with human cases, indicating anthropocentric reporting or high incidence from relatively few animals, although differences in the aggregation of livestock cases limit the inference of spatial synchrony. Controlling HCA requires controlling the disease in livestock (*1*,*2*,*9*) and highlights the need for a One Health approach. Our findings can be used to formulate public health interventions aimed at controlling anthrax in livestock and increasing awareness of the disease, particularly in urban areas. Given the limited resources available, future efforts should focus on high-risk areas for livestock surveillance and control, such as targeted vaccination campaigns (*15*).

Technical Appendix Statistical Methods

## References

[1] Hugh‐Jones M. 1996–97 Global anthrax report. J Appl Microbiol. 1999;87:189–91 . 10.1046/j.1365-2672.1999.00867.x 10475945

[2] World Health Organization. Anthrax in humans and animals: 4th ed. [cited 2013 Feb 10]. http://www.who.int/csr/resources/publications/anthrax_web.pdf

[3] Woods CW, Ospanov K, Myrzabekov A, Favorov M, Plikaytis B, Ashford DA. Risk factors for human anthrax among contacts of anthrax-infected livestock in Kazakhstan. Am J Trop Med Hyg. 2004;71:48–52 . 15238688

[4] Global Infectious Disease and Epidemiology Network. Anthrax in Georgia [cited 2013 Mar 20]. web.gideon.com/web/epidemiology/#

[5] Kracalik IT, Malania L, Tsertsvadze N, Manvelyan J, Bakanidze L, Imnadze P, Evidence of local persistence of human anthrax in the country of Georgia associated with environmental and anthropogenic factors. PLoS Negl Trop Dis. 2013;7:e2388. 10.1371/journal.pntd.0002388 24040426 PMC3764226

[6] Malaniia LO, Imnadze PG, Katsitadze GK, Tsanava SA, Bakanidze LG. Anthrax in Georgia: epidemiological situation and prognosis [in Russian]. Georgian Med News. 2006; (130):67–71 . 16510917

[7] Kulldorff M. A spatial scan statistic. Comm Stat Theory Methods. 1997;26:1481–96. 10.1080/03610929708831995

[8] Hugh-Jones ME, De Vos V. Anthrax and wildlife. Rev Sci Tech. 2002;21:359–83 . 11974621 10.20506/rst.21.2.1336

[9] Doganay M, Metan G. Human anthrax in Turkey from 1990 to 2007. Vector Borne Zoonotic Dis. 2009;9:131–40. 10.1089/vbz.2008.0032 18945187

[10] Özkurt Z, Parlak M, Tastan R, Dinler U, Saglam YS, Ozyurek SF. Anthrax in eastern Turkey, 1992–2004. Emerg Infect Dis. 2005;11:1939–41 . 16485484 10.3201/eid1112.050779 PMC3367647

[11] Chakraborty A, Khan SU, Hasnat MA, Parveen S, Islam MS, Mikolon A, Anthrax outbreaks in Bangladesh, 2009–2010. Am J Trop Med Hyg. 2012;86:703–10. 10.4269/ajtmh.2012.11-0234 22492157 PMC3403762

[12] Maudlin I, Eisler MC, Welburn SC. Neglected and endemic zoonoses. Philos Trans R Soc Lond B Biol Sci. 2009;364:2777–87 and. 10.1098/rstb.2009.0067 19687045 PMC2865085

[13] Kartvelishvili T. Cattle sector and dairy chain developments in Georgia, Azerbaijan and Armenia. In: Peters KJ, Kuipers A, Keane MG, Dimitriadou A, editors. The cattle sector in central and eastern Europe: developments and opportunities in a time of transition. Wageningen (the Netherlands): Wageningen Academic Publishers; 2009. p. 133–52.

[14] Gurbanov S, Akhmedova S. Especially dangerous infections in Azerbaijan. In: O'Connell KP, Skowronski EW, Sulakvelidze A, Bakanidze L, editors. Emerging and endemic pathogens: advances in surveillance, detection and identification. Dordrecht (the Netherlands): Springer; 2010. p. 39–43.

[15] Kracalik IT, Blackburn JK, Lukhnova L, Pazilov Y, Hugh-Jones ME, Aikimbayev A. Analyzing the spatial patterns of livestock anthrax in Kazakhstan in relation to environmental factors: a comparison of local (Gi*) and morphology cluster statistics. Geospat Health. 2012;7:111–26 . 23242686 10.4081/gh.2012.110

