# Supplementary material for: Human Cutaneous Anthrax, Georgia 2010–2012
Source: Emerg Infect Dis. 2014 Feb;20(2):261–4. doi: 10.3201/eid2002.130522 (PMC3901487; doi:10.3201/eid2002.130522)
Supplement: Technical Appendix — Statistical Methods [file 13-0522-Techapp-s1.pdf]

Article DOI: http://dx.doi.org/10.3201/eid2002.130522

Human Cutaneous Anthrax, Georgia 2010–2012

Technical Appendix

Statistical Methods

Negative Binomial Regression

Risk factors for human cutaneous anthrax (HCA), including age, gender, and source of infection, were identified by using a generalized linear model (GLM) regression. Because the data comprised cases stratified by demographic characteristics (Table 1), a count model was used to calculate the incident risk associated with each variable. A GLM with a negative binominal distribution was chosen to calculate incidence risk ratios in SAS v9.2 (SAS Institute, NC, USA; PROC GENMOD). Due to overdispersion in the number of anthrax cases (ratio of the mean/variance was >1) a negative binomial distribution was selected over a Poisson distribution (*1*). The underlying population within each strata of the age groups was adjusted for using the log of the population as an offset in the model. Incidence risk ratios were derived for each variable by exponentiation of the GLM model coefficients.

Empirical Bayes Smoothing

Cumulative incidence per 10,000 population (total cases/median population) were mapped at the community level by using an adjustment for population heterogeneity. Empirical Bayes Smoothing (EBS) in the GeoDa software package (ASU, AZ, USA) (*2*) can be used to adjust for instability in risk estimates caused by small sample sizes and variance in the distribution of cases and the population. The EBS method can be implemented when the numerator data total <3 cases or denominator population data are ≤800, which was the situation in this analysis (*3*). EBS cumulative incidence estimates are derived by using information from the overall study area population mean and the crude cumulative indigence rates. Communities with small sample populations are then adjusted toward the overall mean, and communities with large sample populations undergo very little adjustment (*3*).

Kernel Density Estimation

Kernel density estimation was used to visualize the spatial distribution of cumulative incidence per km^2. Kernel density estimation is a technique for calculating the weighted density of an event over a gridded surface within a kernel, or spatial filter (*4*). Kernel density estimation was performed with the Spatial Analyst Extension for ArcGIS 10. ArcGIS employs the quadratic kernel function described in Silverman (*5*), p. 76, equation 4.5):

$$f(x) = \frac{1}{nh} \sum_{i=1}^{n} K\left(\frac{x - X_i}{h}\right)$$

Where *h* is the bandwidth, *x*-*Xi* is the distance to each village with anthrax *i*. *K* is the quadratic kernel function, which is defined as

$$K(x) = \frac{3}{4}(1 - x^2), |x| \leq 1$$

$$K(x) = 0, x > 1$$

This function was employed to estimate HCA densities using cumulative incidence as the weight. We chose a bandwidth (kernel) of 9.89 km with an output cell size of ≈1 km^2. The resulting output was a map surface representing the cumulative risk for HCA across the country per 1 km^2.

References

1. Hilbe JM. Negative binomial regression. Cambridge (UK): Cambridge University Press; 2011.

2. Anselin L, Syabri I, Kho Y. GeoDa: an introduction to spatial data analysis. Geogr Anal. 2006;38:5–22. http://dx.doi.org/10.1111/j.0016-7363.2005.00671.x

3. Waller LA, Gotway CA. Applied spatial statistics for public health data. Hoboken (NJ): Wiley-Interscience; 2004.

4. Fotheringham AS, Brunsdon C, Charlton M. Quantitative geography: perspectives on spatial data analysis. London: SAGE Publications Ltd; 2000.

5. Silverman BW. Density estimation for statistics and data analysis. Boca Raton (FL): Chapman and Hall/CRC; 1986.
